# Delayed vs. early enteral feeding after repair of congenital recto-vestibular fistula: The effect on perineal wound healing

**DOI:** 10.3389/fped.2022.994249

**Published:** 2023-01-04

**Authors:** Mazen Kurdi, Ahmed Moukhtar, Mahmoud Elkholy, Heidi Alwassia, Maha Bamehriz, Mohammad Gharieb Khirallah

**Affiliations:** ^1^ Peditric Surgery Unit King Abdulaziz University Hospital, King Abdul Aziz University Hospital, Jeddah, Saudi Arabia; ^2^ Faculty of Medicine, Tanta University, Tanta, Egypt

**Keywords:** rectovestibular fistula, perineal wound infection, anterior sagittal anorectoplasty, enteral nutrition, peripheral introduced central line

## Abstract

**Introduction:**

congenital Recto vestibular fistula represents the commonest type of anorectal malformation in females. The treatment of this anomaly is mainly approached either through anterior or posterior sagittal ano-rectoplasty approach. Several perioperative factors may affect the outcome. One of major postoperative complications is the occurrence of wound infection. We aimed to study the effect of delayed vs. early enteral feeding on the occurrence of perineal wound infection (PWI) after repair of congenital recto vestibular fistula.

**Patients and methods:**

Fifty-five infants with recto-vestibular fistula were included. They were managed by single stage anterior sagittal anorectoplasty (ASARP) at an age ≥3 months. Groups A and B included infants who started oral intake on the 6th and 2nd postoperative days respectively. Group A infants were kept on peripheral parenteral nutrition (PPN) during the fasting period.

**Results:**

Superficial wound infection occurred in three cases in group A while it developed in seven cases in group B. Deep perineal infection occurred in two and five cases in group A and group B respectively. The mean hospital stay was 8 days in group A vs. 13 days in group B when PWI developed.

**Conclusion:**

Delayed enteral feeding with PPN keeps the perineal wound less contaminated with stool. This promoted proper and fast healing with lower incidence of PWI. Also, PPN compensates the catabolic effects of both surgical trauma and fasting during the postoperative period and ensures maintenance of normal levels of essential nutrients that allow for proper healing.

## Introduction

Congenital recto-vestibular fistula in females is a common variant of anorectal malformation. This anomaly has the potential for favorable prognosis regarding post-repair sphincter function (1).

There are two options for the management of recto-vestibular fistula, which include posterior sagittal anorectoplasty (PSARP) or anterior sagittal anorectoplasty (ASARP). Also, some surgeons perform a covering colostomy before definitive management, while others perform single-stage repair. Some surgeons plan to repair this anomaly during the neonatal period while other plan to repair the condition after the age of 3 months. Irrespective of the technique used or the timing of repair, good prognosis and functional outcome are anticipated (2–4).

Several perioperative factors may have a potential effect on the prognosis of the repair of recto-vestibular fistula. These factors included antibiotic prophylaxis, mechanical bowel preparation, and nutritional regime. They are characterized by being individually assisted and practiced by surgeons (5).

Nutritional status and regime influence the prognosis of repair of recto-vestibular fistula. Early initiation of enteral feeding is one of the principles of enhanced recovery protocols (ERP) of colorectal surgery in the pediatric age group. However, some surgeons postpone the start of oral intake for 1–2 weeks especially during single stage repair of rectovestibular fistula to avoid local soiling of the perineal wound with stool and to decrease the incidence of wound infection (6).

Perineal wound infection (PWI) is a serious complication. It may lead to failure of the reconstructive surgery, weakness or even degeneration of the sphincter complex and ends in faecal incontinence. Moreover, its healing by fibrosis may lead to stenosis of the new anal opening and massive rectal dilatation that affects bowel evacuation. As a result of this complication the functional outcome will be poor with a bad prognosis. This exerts a psychological burden on parents (7).

We aimed to study the effect of early versus late enteral post-repair feeding on the development of PWI in cases of single stage repair of recto-vestibular fistula.

## Methods

### Study design

This was a randomized control trial. The study included 55 female infants diagnosed with congenital rectovestibular fistula during the period from January 2015 to March 2022. The patients were randomly divided into two groups A and B using opaque sealed envelopes and randomly generated tables. Group A included patients who had delayed initiation of oral intake for 5 days postoperatively. Group B included patients who started oral intake on the 2nd postoperative day and gradually increased as tolerated by the patient. We excluded premature infants, cases with a plan of staged repair and cases with poor nutritional status (their mean level of albumin was 3 gm/dl ± 0.2). All participants were operated on at the age of 3 months or older.

The study was approved by the ethics committee of our institution and assigned code 35054/11/21.

**Figure 1:**
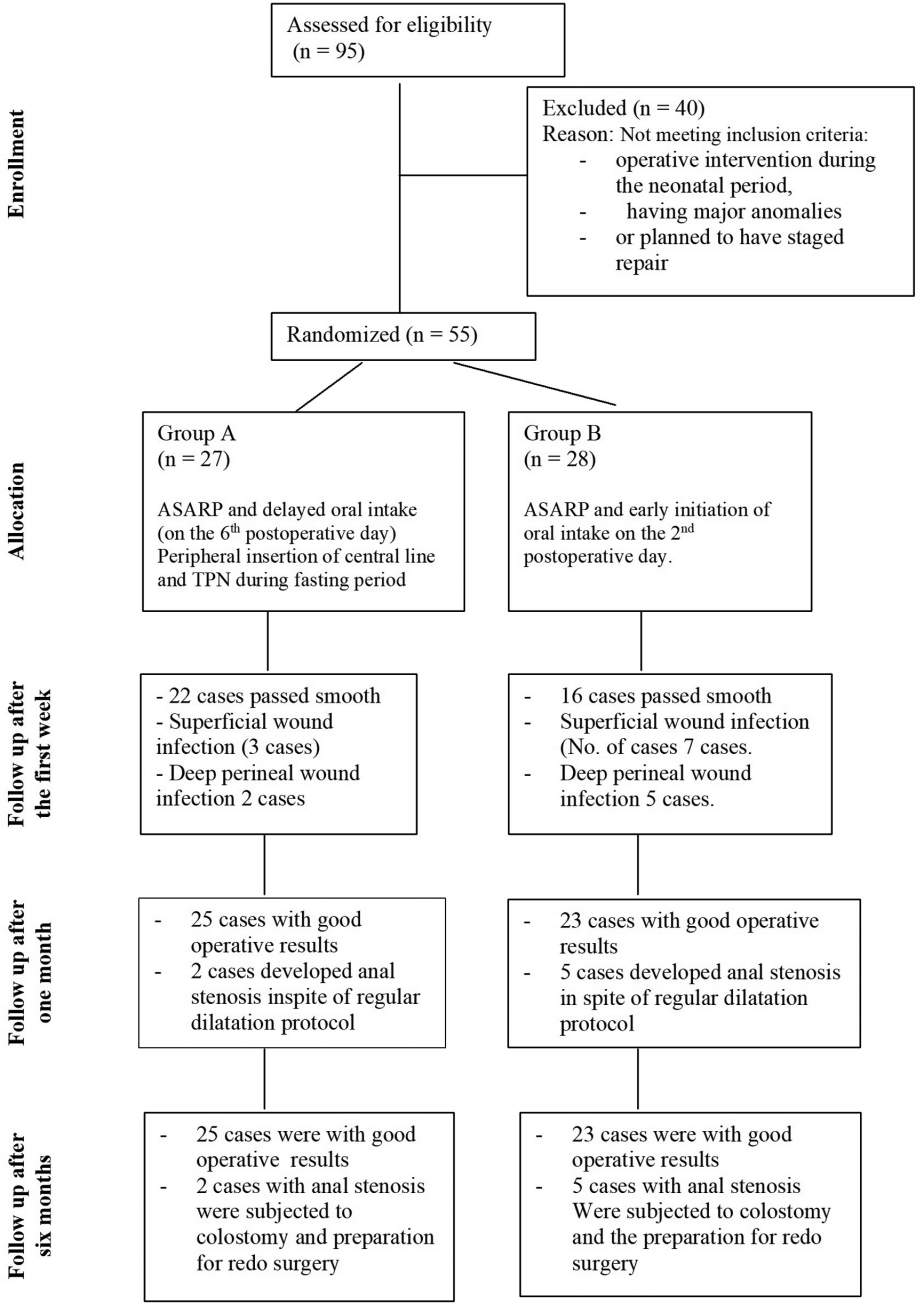
COSORT diagram shoeing the flow of the participants through every stage of the randomized trial inclusion and exclusion criteria of the patients and patients flow chart during the study.

The study was conducted in compliance with COSORT criteria of randomized control trials (Figure 1).

### Preoperative management in both groups

Infants in both groups were subjected to regular dilatation of the fistula with Hegar's dilators twice daily from birth till the time of the operation. The diameter of the dilator gradually increased with the growth of the infant and usually reached size 13 or 14 by the infant's age of 3 months. All infants were admitted to hospital 2 days preoperative. They had mechanical bowel preparation (warm normal saline enema), prophylactic antibiotics, and oral intake of clear fluids. Oral intake was stopped 24 h before surgery and intravenous fluid was initiated. In group A all patients had peripherally introduced central lines (PICL) to be nourished with total parenteral nutrition (TPN) during the first postoperative 5 days.

### Operative management

Infants of both groups had single stage ASARP as definitive treatment. The sphincter saving technique was applied in both groups. Complete urinary diversion using Folly's catheter (size 6 F) was planned in both groups for 5 days postoperatively. The procedures were performed by the expert consultants (author No 1 and author No. 6).

### Postoperative management

Patients of group A were maintained as nothing per os (NPO) for 5 days postoperatively. They were nourished *via* PICL with daily energy requirement provided by TPN. To maintain the efficacy and safety of the PICL, we used the three lumen one and every lumen was identified to a certain medication. Local wound care was performed using local antibiotic cream three times daily. Broad spectrum antibiotic (third generation cephalosporin 100 mg/kg/day intravenous) was administered for 3–5 days. Enteral intake was initiated on the 6th day postoperatively and gradually increased as tolerated by the infant. All infants were followed up every week for the next 6 months.

Patients of group B started oral intake as early as possible according to the protocols of enhanced recovery after colorectal surgery in the pediatric age group. They initiated oral intake on the 2nd postoperative day. These infants started feeding with breast feeding or formula in small amounts and frequent periods, then both the amounts and frequency of periods were increased as the infant tolerated. Local wound care and systemic antibiotics were the same as in group A. The patients were discharged on the sixth day postoperatively. They were followed up according to the same regimen of group A.

### Outcome

The patients were followed up for the development of a PWI. Daily inspection of the wound was performed to detect the signs of PWI. These signs included redness, tenderness, discharge of pus, tender swelling in the wound line and wound dehiscence. The occurrence of some or all these signs in patients of group A or group B, oral intake was restricted and the patient. No complications related to PICL was reported in patients of group A.

### Follow up

All our infants were followed up daily during the first week. The infants who developed PWI continued their treatment in the hospital. Then, there was a regular schedule of follow up every month till 6 months for all cases. We didn't have any cases missed follow-up schedule.

### Statistical analysis

Statistical analysis was conducted using SPSS™ statistical package v. 21 (IBM SPSS, NY, USA). Numerical data were compared using an independent sample t-test, while categorical data were compared using the chi-square test. *p*-values < 0.05 were considered statistically significant. The epidemiologic and characteristic differences between the two study groups were analyzed using chi square.

An *a priori* power analysis was conducted using G*Power version 3.1.9.7 to determine the minimum sample size required to test the study hypothesis. Results indicated the required sample size to achieve 80% power for detecting a medium effect, at a significance criterion of *α* = 0.05, was *N* = 50 for using independent *t* test and chi square to test the hypothesis.

## Results

During the period of study, 95 females with recto-vestibular fistula were admitted for ASARP. Forty cases were excluded due to either having had operative intervention during the neonatal period, having major anomalies or having plans for staged repair. Fifty-five cases met our inclusion criteria.

Inclusion and exclusion criteria of the patients are presented in Figure 1.

Group A included 27 patients who had delayed initiation of oral intake for 5 days postoperatively. Group B included 28 patients who had started oral intake on the 2nd postoperative day and gradually increased in accordance with patient tolerance.

### Preoperative and operative results

There were no significant differences between both groups regarding the mean age at operation (in months), the mean body weight (in kg), and the mean level of albumin (gm/dl) (Table 1).

**Table 1:** Preoperative and operative data in both groups.

	Group A (*N* = 27)	Group B (*N* = 28)	*p*
Age (mean in months)	3.5 (±0.2)	3.7 (±0.4)	0.472
Weight at the operation (mean in kg)	11.3 (±1.3)	10.4 (±1.1)	0.352
Median size of Hegar dilator at the operation	14 (12–15)	13.5 (12–15)	0.463
Albumin level (mean in g/dl)	4.2 (±0.5)	4.3 (±0.4)	0.254
Operative time (mean in minutes)	90 (±15.3)	88 (13.4)	0.138

*p* value is significant if <0.05.

Both groups were operated on using the ASARP approach. No significant differences were found with the regard to the mean operative time (Table 1).

### Postoperative results

Oral intake was initiated in patients of group A on the sixth postoperative day (if there was no PWI) which was statistically significant when correlated to the early initiation of the oral intake in patients of group B.

**Figure 2:**
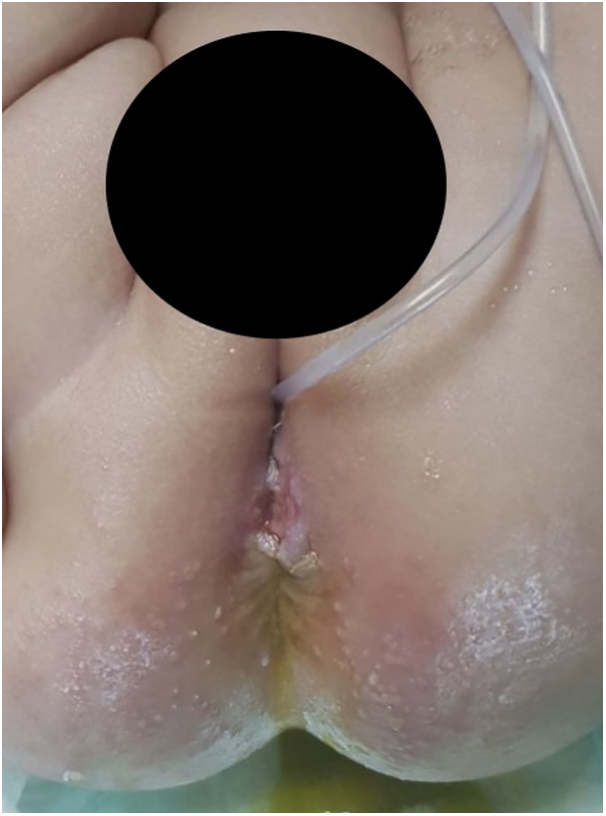
Superficial perineal wound infection.

Superficial wound infection (the infection was limited to the skin and subcutaneous tissue only) developed in three (11%) patients in group A while it developed in seven (25%) patients in group B (Figure 2).

**Figure 3:**
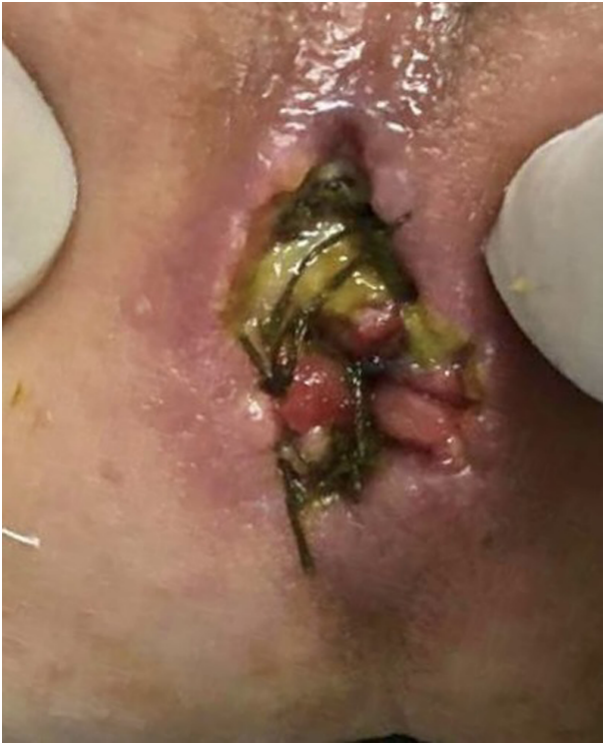
Deep perineal wound infection.

Deep wound infection (the infection extended to the perineal muscles and sphincter with complete disruption of the wound) developed in two (7%) patients in group A while it occurred in five (17.8%) patients in group B and attained statistical significance (*p* value < 0.05) (Figure 3).

In both groups the onset of PWI occurred on the 3rd and 2nd postoperative day respectively, however, the incidence was significantly lower in group A. Superficial wound infection responded to conservative measures such as stop oral intake, swaps for culture and sensitivity, and daily wound care twice using local antibiotics (Garamicin or Fusidine).

The same principles were followed during the management of deep PWI. In group A healing occurred by the eighth day (±2 days) while in group B two cases responded to the conservative measures and healed by the thirteenth day (±2.5 days). This was statistically significant where the *p* value was <0.05. Two cases in group A needed a covering colostomy versus five cases in group B to control PWI that did not respond to the conservative treatment.

During the management of post operative PWI, the mean level of serum albumin in complicated cases of group A was 3.9 gm/dl (±0.3) while it was 3.3 gm/dl (±0.5) in complicated cases of group B. This result was statistically significant (*p* value was <0.05). The mean levels of serum prealbumin in group A was 22 mg/dl (±5 mg/dl) and it was 18 mg/dl (±7 mg/dl) in group B. This was of statistical significance and the *p* value was <0.05 (Table 2).

**Table 2:** Postoperative data in both groups.

	Group A (*N* = 27)	Group B (*N* = 28)	*p* value
Superficial wound infection (No. of cases)	3 (11%)	7 (25%)	0.03*
Deep perineal wound infection	2 (7%)	5 (17.8%)	0.05*
Time of onset of PWI (mean in days ± SD)	3 ± 1	2 ± 1	0.347
Mean serum of albumin in complicated cases	3.9 gm/dl (±0.3)	3.3 gm/dl (±0.5)	0.05*
Mean serum of prealbumin in complicated	22 mg/dl (±5 mg)	18 mg/dl (±7 mg)	0.05*
Hospital stay in uncomplicated cases (mean in days)	7days (±1.2 days)	3 days (±1.1)	0.05*
Readmission to treat PWI (No. of cases)	2 cases (7%)	5 cases (17.8%)	0.03*
Mean hospital stay after readmission (days)	8 days	13 days (±2.5 days)	0.05*

PWI, perineal wound dehiscence and infection. *p* value is significant if <0.05.

* Significant.

### Follow up

In group A regular dilatation with Hegar dilators for 3 months was performed. Two cases developed stenosis. In group B the same protocol was followed but for 5 months and 5 cases remained with stenosis despite the regular dilatation (Table 3).

**Table 3:** Long term follow-up in both groups.

	Group A (*N* = 27)	Group B (*N* = 28)	*p* value
Stenosis	2 (7%)	5 (17.8%)	0.05*
Colostomy	2 (7%)	5 case (17.8%)	0.05*
Mean duration of dilatation (months)	3 ± 0.5	5 ± 0.5	0.05*
Redo surgery	2 (7%)	5 cases (17.8%)	0.05*

*p* value is significant if <0.05.

* Significant.

Redo surgery was planned in two cases (Group A) versus five cases (Group B) (Table 3).

## Discussion

PWI is a serious complication that causes several problems. Apart from affecting the sphincter mechanism and worsening the prognosis post repair of congenital recto-vestibular fistula, it also adds a psychological burden on the parents and increases the cost of hospitalization (8).

The actual incidence of PWI after surgical repair of a rectovaginal fistula is unclear. Several factors may have a direct impact on its development such as poor surgical technique, inadequate preoperative preparation, and improper postoperative care. Some patient factors such as nutritional status and the age of the patient at the time of operation might also contribute (9).

One of the major causes for the development of PWI is the continuous passage of stool over the wound during the early postoperative period. Even with meticulous cleaning and care of the wound, there may be some micro debris of faecal matter that sticks to the sutures and acts as a suitable media for the development of a wound infection.

To avoid this some paediatric surgeons, prefer to operate on females with recto-vestibular fistula during the early hours of an infant's life. The rationale behind this strategy is that the meconium is sterile, thus reducing the incidence of infection. However, this strategy carries the additional risk of early exposure to the anaesthesia and does not allow for proper pre-operative preparation and investigations to detect if other associated anomalies are present (10).

Some pediatric surgeons plan for regular dilatation of the fistula track to avoid ballooning of the rectum and the subsequent discrepancy between the diameter of the pulled through rectum and the future anal opening. This plan allows for the proper assessment of the infant for further associated anomalies, avoids anesthesia-associated-troubles during the early hours of life, and promotes weight gain.

Optimum timing for the initiation of oral intake after ASARP of recto vestibular fistula remains a subject of debate. At present, most centers have their own policy regarding when to initiate the oral feeding. Few reports have discussed this issue either as a case series or in the form of prospective studies. The debate about whether long fasting or early oral intake may be associated with better wound healing is still unsettled (11, 12).

According to the available data, this study represents the first randomized control trial to assess the impact of time of initiation of oral intake in patients with recto-vestibular fistula after ASARP.

In group A, infants were supported by TPN (during the fasting period which lasted 5 days post operatively) supplied through the PICL with the aim of providing adequate calories to overcome the catabolic effect of fasting and surgical trauma and maintain their nutritional status at a proper level to promote healing. We called this procedure “TPN colostomy”, as it helped reduce both the frequency and the volume of intestinal fluids passing over the perineal wound during the early postoperative days. Another advantage of TPN during the early postoperative period was maintaining normal serum levels of albumin, and prealbumin which are required for proper wound healing. The synthesis of these proteins may have been impaired in patients of group B due to inadequate enteral feeding because of pain or as a side effect of drugs in the gastrointestinal tract. There was a decrease in the serum levels of both albumin and prealbumin in patients who developed wound infection in group B. This result attained statistical significance in relation to group A.

The perineal wound was complicated either by superficial or by deep infection. Deep PWI leads to complete perineal dehiscence. PWI developed on the second postoperative day in both groups, with an incidence of 18% in group A and a significantly higher incidence of 42% in group B. This difference may reflect the effect of delayed initiation of oral intake.

The incidence of wound complications in infants in whom early oral intake was initiated ranged from 1% to 27%. Previous studies were either case series or retrospective studies and enrolled only patients that were continuously followed-up at the clinic, including those that were either with ASARP or posterior sagittal anorectoplasty. They also included patients who had colostomies and were operated on either in the neonatal period or in the early infancy. The inclusion of these variables may have been limitations to reach a proper conclusion about the optimal timing of initiation of oral intake (13–15).

The incidence of post operative wound complications reported in previous studies after prolonged fasting ranged from 0% to 22%. These infants were operated on in single stage operations. Moreover, the perioperative variables were not matched in all cases. During the fasting period, the infants were supplied with the daily requirement of fluid and electrolytes and not by TPN (16, 17).

The standards of enhanced recovery protocol after colorectal surgery in children are still evolving. These measures included insurance of early oral intake following colorectal surgery. However, all studies were limited to an age ranging from 2 to 18 years (18).

The main advantage of early initiation of oral intake was the significantly shorter hospital stay seen in group B. However, this was offset by a significantly longer duration of hospital stay on readmission due to PWI in the early post operative period in group B.

In group A, duration of hospital stay was similar to that reported by Menon et al., but another study by Okada et al. reported 4 weeks hospital stay on average (10, 17).

In group B the mean duration of hospital stay was 3 days, which was similar to the duration reported by Upadhyaya et al. and Kulshrestha et al. They documented an average of 2 days in hospital with a range from 2 to 5 days (15, 19).

The sequalae of PWI can cause significant morbidity in infants. The first is healing of the wound by secondary intention and formation of excessive fibrosis that would affect the functioning of both the new anus and the sphincter complex. The new anus may become stenotic if frequent dilatation to overcome this tightness is not carried out. Retention with massive dilatation of the rectum might also develop, necessitating an anoplasty later in their lives. The fibrosis may also affect the sphincter control causing infants to experience a variable degree of soiling. Another complication of PWI is complete dehiscence of the wound with development of an intractable infection that may endanger the sphincter complex. This may result in the need for a colostomy to divert the stool away from the perineum to allow for infection control and healing.

According to our data, stenosis developed in two cases (7%) in group A versus five (17.8%) cases in group B. This result was statistically significant. Two cases in group A underwent colostomy to control infection, while five cases in group B required this type of intervention. The mean duration of dilatation required in group A was 3 months versus a mean of 5 months in group B. This reflected the need for more frequent dilatation in group B to overcome the stenosis after healing of PWI. Due to perineal wound dehiscence and the need of colostomy, two cases in group A required redo surgery versus five cases in group B.

Redo surgery was planned in 3.9%–5.7% in the case series reported by Wakhlu et al. and Kuijper et al. respectively. These were related to the early initiation of oral intake in these patients (13, 20).

On contrary, redo surgery was planned in 0%–2.4% in the series of Menon et al. and Kumar et al. respectively. These patients were managed on the basis of long postoperative fasting period (10, 21).

## Limitations

The small sample size, and the lack of multicenter data are the main limitations. Therefore, a large-scale multicenter study with long-term follow-up is needed to substantiate our proposal.

## Conclusion

Delayed post operative enteral feeding versus early postoperative enteral feeding after ASARP in females with congenital recto-vestibular fistula remains a debated issue. We concluded that delayed enteral feeding with nutritional support by TPN *via* a PICL keeps the perineal wound less contaminated with stool. This promoted proper and fast healing with lower incidence of PWI when compared with infants who were started on oral intake during the early postoperative period. TPN also compensates for the catabolic effect of the surgical trauma and fasting during the postoperative period and ensures the supply of essential nutrients that are necessary for proper healing.

## Data Availability

The raw data supporting the conclusions of this article will be made available by the authors, without undue reservation.
